# Influence of Ripening Stage and Selenium Biofortification on Cherry Tomato Quality During Cold Storage

**DOI:** 10.3390/plants15101562

**Published:** 2026-05-20

**Authors:** Claudio Cannata, Guglielmo Fichera, Anita Ierna, Dimitrios Fanourakis, Rosario Paolo Mauro, Cherubino Leonardi

**Affiliations:** 1 Department of Agriculture, Food and Environment, University of Catania, Via S. Sofia 100, 95123 Catania, Italy; claudio.cannata@unict.it (C.C.); guglielmo.fichera@unict.it (G.F.); cherubino.leonardi@unict.it (C.L.); 2 Institute of BioEconomy, National Research Council (CNR-IBE), Via P. Gaifami 18, 95126 Catania, Italy; anita.ierna@cnr.it; 3 Laboratory of Quality and Safety of Agricultural Products, Landscape and Environment, Department of Agriculture, School of Agricultural Sciences, Hellenic Mediterranean University, Estavromenos, 71004 Heraklion, Greece; dfanourakis@hmu.gr

**Keywords:** selenium deficiency, biofortification, fresh product, shelf life, functional traits

## Abstract

Preharvest selenium (Se) biofortification is a promising strategy to enhance both the nutritional value and postharvest performance of vegetables. However, its effects on cherry tomato quality during storage, particularly in relation to ripening stage at harvest, remain poorly understood. This study evaluated the impact of foliar Se application (0.5 mM, as Na_2_SeO_4_) on carpometric, compositional, and functional traits of cherry tomatoes harvested at two ripening stages (orange-red and deep red) and stored for 0, 10, and 20 days at 11.0 ± 0.5 °C. The Se application increased fruit Se concentration (∼30-fold) and improved dry matter (+8.1%) and firmness (+8.3%) throughout storage. At the end of storage, all fruits showed reduced firmness (up to −44%) and increased fresh weight loss (up to 8.5%), although Se-biofortified fruits consistently maintained a higher dry matter content. The effects of Se on compositional traits were ripening stage-dependent, as it enhanced glucose (+8.2%), fructose (+10.0%), and total sugars (+9.4%) in fully ripe fruits, while increasing titratable acidity in less mature ones (+8.2%). Moreover, Se reduced total carotenoids in fully ripe fruits (−13.2%) but increased ascorbic acid during storage (+19.4%), irrespective of ripening stage. Overall, Se biofortification effectively enriched cherry tomatoes and modulated their postharvest behavior. However, the contrasting, stage-dependent effects of Se biofortification on the functional compounds of cherry tomatoes emphasize the need to refine the biofortification strategy in order to achieve a more consistent and comprehensive improvement in fruit quality.

## 1. Introduction

Tomato (*Solanum lycopersicum* L.) is the second most important vegetable crop worldwide after potato, being cultivated on ~5 Mha [1]. In Southern Italy, where most of the national greenhouse tomato area is concentrated (5.92 out of 8.59 kha) [2], cherry tomato is among the predominant cultivated typologies, being appreciated by consumers because of its distinctive organoleptic properties. Nowadays, tomato consumption is considered a key indicator of healthy dietary patterns, owing to its significant contribution to human diet in terms of carotenoids, (poly)phenols, vitamins, and minerals [3]. From a postharvest perspective, tomatoes are climacteric fruits, hence their ripening and senescence processes continue even after detachment from the plant [4]. This physiological behavior leads to progressive modifications of their initial appearance and compositional profile, with significant visual and biochemical deterioration occurring up to a state where the fruits become valueless for consumers [5]. For this reason, beyond refrigerated storage, tomato producers often harvest fruits at maturity stages preceding full ripeness, in order to extend their postharvest life, regulate market supply, and minimize postharvest losses [6]. Accordingly, in tomato, the maturity stage at harvest is a key determinant of overall fruit quality (e.g., epicarp color, carotenoid content, sugars, and ascorbic acid), along with of the ability of these attributes to withstand storage conditions, hence influencing quality evolution and shelf life during cold storage [3,7]. On the other hand, tomato has been the focus of extensive efforts aimed at extending postharvest life, including breeding strategies, advanced packaging systems, or the adoption of preharvest interventions to modulate the postharvest metabolism, with the final aim of maintaining the highest quality standards between production and consumption [8,9]. Within this framework, in recent years increasing interest has been directed toward the preharvest application of selenium (Se) in tomato (mineral biofortification), owing to the effects of this element on both human health and the metabolism of horticultural products [10,11,12,13]. From a nutritional perspective, Se has gained attention as an essential component of selenoproteins, which are involved in a broad range of physiological functions, including antioxidant defense, T-cell-mediated immunity, thyroid hormone metabolism, immune regulation, and redox homeostasis [14,15,16]. Despite this biological relevance, Se deficiency remains a widespread nutritional concern in many regions of the world where its availability in soils is inherently low. Moreover, predictive models indicate that ongoing climate change may further decrease soil Se concentrations across more than half of the global land surface, hence exacerbating the Se deficiency in human diets [17].

Beyond its effects on tomato fruit quality [11,18,19], emerging evidence suggests that Se can also influence postharvest physiology, hence contributing to tomato shelf-life extension. For instance, Puccinelli et al. [20] reported reduced respiration and ethylene production in Se-biofortified tomatoes during postharvest ripening, an effect consistent with the downregulation of ethylene biosynthesis genes induced by preharvest foliar Se application reported by Zhu et al. [21]. These effects, together with the Se-induced enhancement of the antioxidant system, which limits reactive oxygen species (ROS) accumulation, contribute to delayed fruit senescence during tomato postharvest life. However, to date, studies investigating the effects of Se biofortification on tomato postharvest physiology remain fragmented, with several aspects still requiring clarification. This is particularly evident when considering its impact on the quality and shelf life of cherry tomatoes harvested at different ripening stages, as typically occurs under commercial conditions. Elucidating the stage-dependent effects of Se biofortification on tomato quality and postharvest performance could provide insights for developing strategies to maximize the overall fruit quality, thereby meeting the growing demand for nutrient-dense products and efficient agri-food supply chains. For these reasons, the present study investigated the effects of preharvest foliar Se application on key quality traits of cherry tomatoes subjected to refrigerated storage. The Se-biofortified fruits were harvested at two ripening stages (i.e., orange-red and deep red), and the evolution of their compositional, compositional and functional traits was assessed at harvest and after 10 and 20 days of refrigerated storage, in comparison to untreated fruits.

## 2. Results

**Figure 1:**
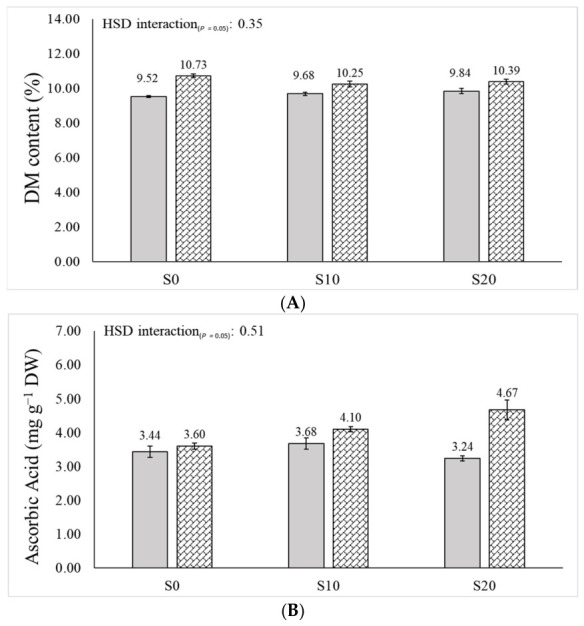
Fruit dry matter (**A**), and ascorbic acid (**B**) content of cherry tomatoes as affected by ”treatment × storage time” interaction (mean ± standard error). Solid gray bars: control; hatched bars: Se-treated.

The significance resulting from the ANOVA related to the main factors and their interactions is reported in Table 1 (Fisher–Snedecor *F*-test), whereas their effects on variable means are reported in Table 2, Table 3, Table 4, Table 5 and Table 6 and Figure 1.

### 2.1. Carpometric Traits and Selenium Content

Fruit harvested at stage G had higher FW (12.1 g, +17.5%), L (29.1 mm, +9.0%), and D (27.4 mm, +8.7%) than those harvested at stage E (10.3 g, 26.7 mm, and 25.2 mm, respectively) (Table 2). The Se application did not influence the main fruit carpometric traits (i.e., fruits FW, L, and D) (Table 2). Differently, the Se-treated fruits had a higher Se concentration (13.34 µg 100 g^−1^ FW, +3002%) than the control fruits (0.43 µg 100 g^−1^ FW) (Table 2).

Overall, the stage G fruits proved to have the highest values of DM content (10.27%) and FWL (7.04%) (Table 3). Regardless of ripening stage and storage time, Se-treated fruits showed higher DM content (10.46%) and firmness (4.58 N, +8.3%) than the control (9.68% and 4.23 N, respectively) (Table 3). Moreover, the ANOVA revealed that the Se-treated fruits showed higher DM content than the controls throughout the storage period, although a significant increase between S_0_ and S_20_ (i.e., from 9.52% to 10.39%) was noted in the control fruits (Figure 1A). The significant ”ripening stage × storage time” interaction further highlighted as fruits harvested at stage E experienced a decline in DM content passing from S_0_ to S_10_ (from 10.08 to 9.72%), and a progressive firmness loss, with the steepest decrease occurring in the late storage period, i.e., between S_10_ and S_20_ (from 4.73 to 2.91 N, −38.5%). Differently, stage G fruits showed stable DM content values throughout the storage period, and the most significant firmness decline in the early storage period, i.e., from S_0_ to S_10_ (from 5.74 to 3.88 N, −32.4%) (Table 3). At the same time, fruit FWL increased during storage, with fruits harvested at stage G showing higher values than those at harvest stage E, mostly at S_20_ (9.24 vs. 7.77%) (Table 3).

Fruits harvested at stage E showed the highest L* (35.1), a* (20.7), and b* values (22.0), along with the lowest TCI (39.1). Moreover, this last variable experienced a significant increase passing from S_0_ to S_10_ (from 38.3 to 40.0) (Table 4). These chromatic variables were not influenced by Se applications and showed a distinctive temporal trend in relation to the ripening stage. Specifically, over the S_0_–S_20_ period, the stage G fruits showed the strongest decline in L* (from 35.3 to 34.0), a* (from 20.6 to 19.8), and b* (from 21.9 to 20.8) values (Table 4). Differently, fruits harvested at stage E displayed positive variation in a* values (from 19.8 to 21.4) and stable b* values (22.0 on average).

### 2.2. Compositional Traits

The stage E fruits had lower levels of D-glucose, D-fructose, and TSC compared to those harvested at stage G (Table 5). Moreover, the ANOVA revealed significant ”ripening stage × treatment” and ”ripening stage × storage time” interactions for all these variables. Accordingly, only in stage G fruits the Se application promoted D-glucose (207 vs. 224 mg g^−1^ DW, +8.2%), D-fructose (219 vs. 241 mg g^−1^ DW, +10.0%), and TSC (425 vs. 465 mg g^−1^ DW, +9.4%) compared to control fruits (Table 5). Moreover, over the S_0_–S_20_ period, stage G fruits experienced the most marked declines in D-glucose (from 240 to 200 mg g^−1^ DW, −16.6%), D-fructose (from 251 to 212 mg g^−1^ DW, −15.5%), and TSC (from 492 to 412 mg g^−1^ DW, −16.3%) (Table 5). Despite these reductions, at S_20_, these fruits showed higher D-glucose (200 mg g^−1^ DW), D-fructose (212 mg g^−1^ DW), and TSC (412 mg g^−1^ DW) contents than those harvested at stage E (175, 189, and 365 mg g^−1^ DW, respectively) (Table 5). Regarding TA and TSSs/TA significant ”ripening stage × treatment” and ”ripening stage × storage time” interactions were detected (Table 1). Overall, stage E fruits proved higher TA (0.71% CAEs) and lower TSSs/TA values (9.53) than stage G fruits (0.59% CAEs and 12.70, respectively) (Table 5). The biofortification treatment significantly increased TA values, but only in stage E fruits (+8.2%). Consequently, in control fruits the TSSs/TA ratio passed from 9.84 (Stage E fruits) to 12.41 (stage G fruits, +26.1%), whereas it rose from 9.22 (stage E) to 13.00 (stage G) in Se-treated ones (+40.1%) (Table 5). Irrespective of treatment, TA and TSSs/TA remained stable throughout the storage period in stage E fruits (0.712 and 9.53, on average for storage time, respectively). Differently, in fruits harvested at stage G, TA increased from S_0_ to S_10_ (from 0.524 to 0.613, +17.0%), along with a consequent decline in TSSs/TA ratio (from 14.54 to 12.01, −17.4%) (Table 5).

### 2.3. Functional Traits

As reported in Table 1, TPC and AA were exclusively influenced by ripening stage and storage time. Specifically, stage E fruits showed higher TPC and AA values (6.91 mg GAE g^−1^ DW and 7.70 mg TEs g^−1^ DW, respectively) than stage G fruits (6.36 mg GAE g^−1^ DW and 7.41 mg TEs g^−1^ DW, respectively) (Table 6). On the other hand, over the storage period, both TPC and AA peaked at S_10_ (6.97 mg GAE g^−1^ DW and 8.01 mg TEs g^−1^ DW, respectively) and then declined at S_20_ (6.51 mg GAE g^−1^ DW and 7.45 mg TEs g^−1^ DW, respectively) (Table 6). Compared to the control, the Se treatment reduced TCC values in stage G fruits (1096 vs. 968 µg g^−1^ DW, −13.2%). Concurrently, as storage progressed, stage E fruits showed a gradual increase in TCC (from 979 to 1206 µg g^−1^ DW, +23.2%), while stage G fruits displayed a significant TCC reduction from S_0_ to S_10_ (from 1095 to 977 µg g^−1^ DW, −10.8%) (Table 6). Irrespective of the storage time, ascorbic acid peaked in Se-treated fruits harvested at stage E (4.24 mg g^−1^ DW). Across ripening stages, ascorbic acid remained stable in control fruits over the S_0_ – S_20_ period, while it increased in the treated fruits (from 3.60 to 4.67 mg g^−1^ DW, +29.7%) (Figure 1B). Regardless foliar treatment, ascorbic acid increased markedly from S_0_ to S_20_ in fruits harvested at stage E (from 3.31 to 4.11 mg g^−1^ DW, +24.2%), while no significant variations were recorded in fruits harvested at stage G.

### 2.4. Correlation Among Variables

Overall, 120 correlations were analyzed, of which 35 (29% of the total) were significant, highlighting 19 negative and 16 positive relationships (Table 7). Among the negative correlations, the strongest associations were found between TA and TSSs/TA ratio (−0.968 ***), L* and TCI values (−0.934 ***), firmness and FWL (−0.744 ***) firmness and TCI (−0.733 ***), and between DPPH and FWL (−0.679 ***) (Table 7). Additional further negative relationships were found between TA and TSC (−0.680 ***), D-fructose (−0.674 ***), and D-glucose (−0.659 ***). Conversely, the strongest positive correlations were detected between TSC and D-fructose (0.982 ***), TSC and D-glucose (0.981 ***), D-glucose and D-fructose (0.925 ***), a* and b* (0.823 ***), and D-fructose and the TSSs/TA ratio (0.708 ***). Other notable positive correlations included those between TSC and the TSSs/TA ratio (0.706 ***), DPPH and TPC (0.667 ***), TCC and FWL (0.643 ***), firmness and L* (0.609 ***), and firmness and D-glucose (0.561 ***).

### 2.5. Tables and Figures

**Table 1:** Fisher–Snedecor *F*-values of the main factors and their interactions related to the observed variables, with the significance resulting from the ANOVA.

	Ripening Stage (R)	Se Treatment (T)	Storage Time (S)	R × T	R × S	T × S	R × T × S
Average FW	17.2 **	NS	-	NS	-	-	-
L	43.22 **	NS	-	NS	-	-	-
D	28.9 **	NS	-	NS	-	-	-
Se content	NS	1180.0 ***	-	NS	-	-	-
DM content	36.8 ***	139.3 ***	NS	NS	6.0 ***	11.2 ***	NS
Firmness	NS	5.8 *	106.9 ***	NS	4.5 *	NS	NS
FWL	80.1 ***	NS	1160.7 ***	NS	13.5 ***	NS	NS
L*	46.2 ***	NS	75.2 ***	NS	10.9 **	NS	NS
a*	10.9 **	NS	NS	NS	22.7 ***	NS	NS
b*	20.8 ***	NS	NS	NS	10.4 ***	NS	NS
TCI	30.4 ***	NS	79.5 ***	NS	NS	NS	NS
Glucose	74.8 ***	6.1 *	47.0 ***	11.2 **	3.5 *	NS	NS
Fructose	104.5 ***	5.2 *	42.4 ***	34.0 ***	3.6 *	NS	NS
Total Sugars	102 ***	6.5 *	51.1 ***	23.8 ***	3.9 *	NS	NS
TA	107.5 ***	6.2 *	6.7 **	5.5 *	14.1 ***	NS	NS
TSSs/TA	169.2 ***	NS	7.1 **	6.1 *	24.4 ***	NS	NS
TCC	NS	32.6 ***	37.1 ***	6.1 *	11.8 ***	NS	NS
TPC	25.3 ***	NS	9.3 **	NS	NS	NS	NS
Ascorbic Acid	NS	50.0 ***	8.1 **	15.3 ***	5.8 **	16.4 ***	NS
DPPH	6.9 *	NS	17.7 ***	NS	NS	NS	NS

FW: fresh weight; DM: dry matter; FWL: fresh weight loss; TA: titratable acidity; TSSs: total soluble solids; TCC: total carotenoids content; TPC: total phenolic content; NS: not significant; *, ** and ***: HSD value significant at *p* = 0.05, 0.01 and 0.001, respectively.

**Table 2:** Main carpometric traits and Se content at S_0_ of cherry tomatoes (mean ± standard error).

Source of Variation	Fruit FW (g)	L (mm)	D (mm)	Se Content (µg 100 g^−1^ FW)
Ripening Stage (R)				
Stage E	10.3 ± 0.2 b	26.7 ± 0.3 b	25.2 ± 0.4 b	6.66 ± 2.80 a
Stage G	12.1 ± 0.5 a	29.1 ± 0.4 a	27.4 ± 0.3 a	7.11 ± 2.99 a
				
Se Treatment (T)				
Control	10.8 ± 0.3 a	27.6 ± 0.6 a	25.9 ± 0.4 a	0.43 ± 0.02 b
Treated	11.5 ± 0.6 a	28.2 ± 0.5 a	26.7 ± 0.6 a	13.34 ± 0.37 a
T × R	NS	NS	NS	NS
Overall mean	11.2	27.9	26.3	6.89

Different letters within each column’s factor indicate significance at Tukey’s HSD test (*p* ≤ 0.05). NS: not significant. FW: fresh weight.

**Table 3:** Main carpometric variables of cherry tomatoes as affected by the main factors (mean ± standard error). The ”ripening stage × Se treatment” and ”ripening stage × storage time” interactions are also reported.

	Se Treatment		Storage Time			Ripening Stage Mean
	Control	Treated	S_0_	S_10_	S_20_	
DM content (%)						
Stage E	9.49 ± 0.04	10.25 ± 0.12	10.08 ± 0.28	9.72 ± 0.12	9.84 ± 0.14	9.87 ± 0.11 b
Stage G	9.88 ± 0.10	10.66 ± 0.09	10.17 ± 0.28	10.22 ± 0.17	10.42 ± 0.12	10.27 ± 0.11 a
Mean	9.68 ± 0.07 b	10.46 ± 0.09 a	10.12 ± 0.19 a	9.97 ± 0.13 a	10.12 ± 0.13 a	
HSD interaction (*p* = 0.05)	NS		0.35			
			
Firmness (N)						
Stage E	4.38 ± 0.38	4.68 ± 0.41	5.71 ± 0.27	4.73 ± 0.14	2.91 ± 0.11	4.53 ± 0.27 a
Stage G	4.09 ± 0.39	4.48 ± 0.41	5.74 ± 0.23	3.88 ± 0.09	3.15 ± 0.21	4.28 ± 0.28 a
Mean	4.23 ± 0.27 b	4.58 ± 0.28 a	5.73 ± 0.17 a	4.30 ± 0.15 b	3.19 ± 0.12 c	
HSD interaction (*p* = 0.05)	NS		0.77			
			
FWL (%)						
Stage E	6.01 ± 0.65	6.00 ± 0.65	-	4.24 ± 0.04	7.77 ± 0.10	6.01 ± 0.44 b
Stage G	7.10 ± 0.85	6.99 ± 0.94	-	4.85 ± 0.12	9.24 ± 0.37	7.04 ± 0.61 a
Mean	6.55 ± 0.53 a	6.50 ± 0.57 a	-	4.55 ± 0.11 b	8.50 ± 0.33 a	
HSD interaction (*p* = 0.05)	NS		0.48			

Different letters among means of each main factor indicate significance in Tukey’s HSD test (*p* ≤ 0.05). NS: not significant; DM: dry matter; FWL: fresh weight loss.

**Table 4:** Main chromatic variables of cherry tomatoes as affected by the main factors (mean ± standard error). The ”ripening stage × Se treatment” and ”ripening stage × storage time” interactions are also reported.

	Se Treatment		Storage Time			Ripening Stage Mean
	Control	Treated	S_0_	S_10_	S_20_	
L* (relative units)						
Stage E	35.0 ± 0.1	35.1 ± 0.2	35.5 ± 0.1	34.6 ± 0.1	35.0 ± 0.1	35.1 ± 0.1 a
Stage G	34.5 ± 0.3	34.6 ± 0.2	35.3 ± 0.1	34.2 ± 0.2	34.0 ± 0.1	34.5 ± 0.2 b
Mean	34.7 ± 0.2 a	34.9 ± 0.1 a	35.4 ± 0.1 a	34.4 ± 0.1 b	34.5 ± 0.2 b	
HSD interaction (*p* = 0.05)	NS		0.4			
			
a* (relative units)						
Stage E	20.6 ± 0.2	20.8 ± 0.3	19.8 ± 0.1	20.9 ± 0.1	21.4 ± 0.1	20.7 ± 0.2 a
Stage G	20.1 ± 0.2	20.3 ± 0.2	20.6 ± 0.1	20.2 ± 0.3	19.8 ± 0.1	20.5 ± 0.1 b
Mean	20.3 ± 0.2 a	20.5 ± 0.2 a	20.2 ± 0.1 a	20.6 ± 0.2 a	20.6 ± 0.3 a	
HSD interaction (*p* = 0.05)	NS		0.4			
					
b* (relative units)						
Stage E	21.8 ± 0.1	22.1 ± 0.1	21.7 ± 0.1	22.0 ± 0.1	22.1 ± 0.1	22.0 ± 0.1 a
Stage G	21.3 ± 0.3	21.5 ± 0.2	21.9 ± 0.1	21.4 ± 0.3	20.8 ± 0.2	21.8 ± 0.2 b
Mean	21.5 ± 0.1 a	21.8 ± 0.1 a	21.8 ± 0.1 a	21.7 ± 0.2 a	21.5 ± 0.2 a	
HSD interaction (*p* = 0.05)	NS		0.7			
						
TCI						
Stage E	39.2 ± 0.4	39.0 ± 0.4	37.9 ± 0.2	39.8 ± 0.1	39.6 ± 0.3	39.1 ± 0.2 b
Stage G	39.9 ± 0.4	39.7 ± 0.2	38.8 ± 0.2	40.1 ± 0.2	40.5 ± 0.1	39.8 ± 0.2 a
Mean	39.6 ± 0.3 a	39.3 ± 0.2 a	38.3 ± 0.2 b	40.0 ± 0.1 a	40.0 ± 0.2 a	
HSD interaction (*p* = 0.05)	NS		NS			

Different letters among means of each main factor indicate significance in Tukey’s HSD test (*p* ≤ 0.05). NS: not significant; TCI: tomato color index.

**Table 5:** Compositional traits of cherry tomatoes as affected by the main factors (mean ± standard error). The ”ripening stage × Se treatment” and ”ripening stage × storage time” interactions are also reported.

	Se Treatment		Storage Time			Ripening Stage Mean
	Control	Treated	S_0_	S_10_	S_20_	
D-Glucose (mg g^−1^ DW)						
Stage E	191 ± 5	188 ± 4	204 ± 5	188 ± 2	175 ± 2	189 ± 3 b
Stage G	207 ± 6	224 ± 8	240 ± 8	205 ± 3	200 ± 4	215 ± 5 a
Mean	199 ± 4 b	206 ± 6 a	222 ± 7 a	197 ± 3 b	188 ± 4 b	
HSD interaction (*p* = 0.05)	12		16			
			
D-Fructose (mg g^−1^ DW)						
Stage E	206 ± 4	196 ± 3	212 ± 4	203 ± 4	189 ± 2	201 ± 3 b
Stage G	219 ± 5	241 ± 7	251 ± 9	226 ± 4	212 ± 5	229 ± 5 a
Mean	212 ± 4 b	219 ± 7 a	232 ± 7 a	215 ± 5 b	201 ± 4 c	
HSD interaction (*p* = 0.05)	11		15			
			
TSC (mg g^−1^ DW)						
Stage E	397 ± 9	385 ± 7	416 ± 8	391 ± 5	365 ± 4	391 ± 6 b
Stage G	425 ± 11	465 ± 15	492 ± 17	432 ± 6	412 ± 10	445 ± 10 a
Mean	411 ± 8 b	425 ± 13 a	454 ± 14 a	411 ± 7 b	388 ± 9 c	
HSD interaction (*p* = 0.05)	21		15			
			
TA (% CAEs)						
Stage E	0.68 ± 0.01	0.74 ± 0.01	0.73 ± 0.02	0.70 ± 0.01	0.71 ± 0.02	0.71 ± 0.01 a
Stage G	0.59 ± 0.02	0.59 ± 0.02	0.52 ± 0.01	0.61 ± 0.02	0.64 ± 0.02	0.59 ± 0.01 b
Mean	0.64 ± 0.02 b	0.67 ± 0.02 a	0.63 ± 0.03 b	0.66 ± 0.02 ab	0.68 ± 0.02 a	
HSD interaction (*p* = 0.05)	0.05		0.06			
						
TSSs/TA (relative units)						
Stage E	9.84 ± 0.18	9.22 ± 0.21	8.97 ± 0.23	9.77 ± 0.23	9.84 ± 0.22	9.53 ± 0.15 b
Stage G	12.41 ± 0.53	13.00 ± 0.55	14.54 ± 0.34	12.01 ± 0.28	11.56 ± 0.46	12.70 ± 0.38 a
Mean	11.13 ± 0.41 a	11.11 ± 0.54 a	11.76 ± 0.86 a	10.89 ± 0.38 b	10.70 ± 0.36 b	
HSD interaction (*p* = 0.05)	0.95		1.31	1.32		

Different letters among means of each main factor indicate significance in Tukey’s HSD test (*p* ≤ 0.05). DW: dry weight; TSC: total sugars content; TA: titratable acidity; TSSs: total soluble solids.

**Table 6:** Functional variables of cherry tomatoes as affected by the main factors (mean ± standard error). The ”ripening stage × treatment” and ”ripening stage × storage time” interactions are also reported.

	Treatment		Storage Time			Ripening Stage Mean
	Control	Treated	S_0_	S_10_	S_20_	
TPC (mg GAE g^−1^ DW)						
Stage E	7.00 ± 0.19	6.82 ± 0.06	6.73 ± 0.17	7.27 ± 0.19	6.74 ± 0.05	6.91 ± 0.1 a
Stage G	6.28 ± 0.14	6.44 ± 0.14	6.12 ± 0.09	6.66 ± 0.16	6.29 ± 0.18	6.36 ± 0.1 b
Mean	6.64 ± 0.15 a	6.63 ± 0.09 a	6.42 ± 0.13 b	6.97 ± 0.15 a	6.51 ± 0.11 b	
HSD interaction (*p* = 0.05)	NS		NS			
			
TCC (µg g^−1^ DW)						
Stage E	1096 ± 36	1040 ± 39	979 ± 27	1019 ± 23	1206 ± 13	1068 ± 27 a
Stage G	1088 ± 30	968 ± 27	1095 ± 45	977 ± 34	1012 ± 31	1028 ± 26 a
Mean	1092 ± 23 a	1004 ± 24 b	1037 ± 30 ab	998 ± 20 b	1109 ± 19 a	
HSD interaction (*p* = 0.05)	67		82			
			
Ascorbic acid (mg g^−1^ DW)						
Stage E	3.20 ± 0.07	4.24 ± 0.27	3.31 ± 0.13	3.74 ± 0.18	4.11 ± 0.49	3.72 ± 0.18 a
Stage G	3.70 ± 0.12	4.01 ± 0.12	3.73 ± 0.08	4.04 ± 0.12	3.80 ± 0.22	3.86 ± 0.09 a
Mean	3.45 ± 0.09 b	4.12 ± 0.14 a	3.52 ± 0.1 b	3.89 ± 0.11 a	3.95 ± 0.26 a	
HSD interaction (*p* = 0.05)	0.37		0.51			
			
DPPH (mg TEs g^−1^ DW)						
Stage E	7.62 ± 0.22	7.79 ± 0.18	7.26 ± 0.18	8.30 ± 0.22	7.54 ± 0.06	7.70 ± 0.14 a
Stage G	7.45 ± 0.12	7.37 ± 0.12	7.16 ± 0.12	7.71 ± 0.13	7.35 ± 0.10	7.41 ± 0.08 b
Mean	7.53 ± 0.12 a	7.58 ± 0.12 a	7.21 ± 0.1 b	8.01 ± 0.15 a	7.45 ± 0.06 b	
HSD interaction (*p* = 0.05)	NS		NS			

Different letters among means of each main factor indicate significance in Tukey’s HSD test (*p* ≤ 0.05). NS: not significant; DW: dry weight; TPC: total phenolic content; TCC: total carotenoid content.

**Table 7:** Pearson product-moment correlation coefficient values (r) among variables.

	DM Content	Firmness	FWL	L*	a*	b*	TCI	D-Glucose	D-Fructose	TSC	TA	TSSs/TA	TPC	TCC	Ascorbic Acid
Firmness	NS	—													
FWL	NS	−0.744 ***	—												
L	NS	0.609 ***	NS	—											
a*	NS	NS	NS	NS	—										
b*	NS	NS	NS	0.671 ***	0.823 ***	—									
TCI	NS	−0.733 ***	NS	−0.934 ***	NS	−0.499 **	—								
D-Glucose	NS	0.561 ***	NS	NS	NS	NS	NS	—							
D-Fructose	NS	0.456 **	NS	NS	NS	NS	NS	0.925 ***	—						
TSC	NS	0.517 **	NS	NS	NS	NS	NS	0.981 ***	0.982 ***	—					
TA	NS	NS	NS	NS	NS	NS	NS	−0.659 ***	−0.674 ***	−0.680 ***	—				
TSSs/TA	NS	NS	NS	NS	NS	NS	NS	0.678 ***	0.708 ***	0.706 ***	−0.968 ***	—			
TPC	NS	NS	−0.572 **	NS	NS	NS	NS	−0.442 **	−0.358 *	−0.407 *	0.502 **	−0.518 **	—		
TCC	−0.375 *	−0.472 **	0.643 ***	NS	0.339 *	NS	NS	NS	NS	NS	NS	NS	NS	—	
Ascorbic Acid	NS	NS	NS	NS	NS	NS	NS	NS	NS	NS	NS	NS	NS	NS	—
DPPH	NS	NS	−0.679 ***	NS	NS	NS	NS	−0.432 **	−0.331 *	−0.389 *	NS	NS	0.667 ***	NS	NS

FW: fresh weight; DM: dry matter; FWL: fresh weight loss; TA: titratable acidity; TSSs: total soluble solids; TCC: total carotenoids content; TPC: total phenolic content; NS: not significant; *, **, and ***: significant at *p* ≤ 0.05, 0.01, and 0.001, respectively.

## 3. Discussion

Under our experimental conditions, carpometric traits at harvest were exclusively influenced by ripening stage, reflecting the typical variations associated with fruit ripening [22]. On the other hand, the ~30-fold increase in fruit Se concentration highlights the effectiveness of the applied treatments in promoting the Se uptake and translocation to the edible tissues. These findings are consistent with previous reports [18,23], confirming that Se biofortification in tomato can be effectively achieved through foliar applications without affecting fruit size or weight, even under a strong increase in fruit Se content. Similar enhancements in tomato Se content have been reported by other authors [12,19,24,25], although the extent of accumulation may vary depending on genotype, environmental conditions, and application protocols [26,27]. From a nutritional perspective, the Se content achieved in this study (13.34 µg 100 g^−1^ FW) represents a relevant contribution, as a 100 g portion of Se-biofortified tomatoes would cover 19% of the daily adequate intake established by European Food Safety Authority (70 µg day^−1^), while remaining below the tolerable upper intake level (255 µg day^−1^) [14,28]. Since adequate micronutrient intakes are best achieved through a varied diet rich in vegetables, fruits, and berries, Se-biofortified tomatoes should be regarded as one valuable component of a broader dietary strategy, in which different biofortified plant-based foods may collectively improve nutrient adequacy and phytochemical diversity while addressing specific consumer needs.

Beyond its nutritional role, Se biofortification has been reported to influence tomato quality and shelf life [12,19,24].

Ripening stage and storage time significantly influenced the postharvest behavior of tomatoes. Fruits harvested at the earliest stage proved a more gradual decline in firmness, whereas fully ripe fruits showed a more marked reduction during the early storage phase. These patterns are consistent with the climacteric nature of tomato, where less mature fruits undergo progressive softening, while fully ripe fruits are more susceptible to rapid cell wall degradation [29,30]. Similarly, fresh weight loss increased during storage and was higher in fruits harvested at the deep red stage, in agreement with previous findings indicating higher transpiration and respiration rates in more mature fruits [31,32]. However, Se application enhanced tomato dry matter content and firmness, which remained significantly higher than those of the control group throughout storage. This effect is likely attributable to Se-enhanced carbon assimilation and its partitioning within the plant, together with a reduction in the fruit respiration rate during storage [19,20,33]. Moreover, the higher firmness observed in Se-treated fruits suggests that Se may delay softening through partial suppression of ethylene biosynthesis and improved membrane stability [21,34]. Indeed, as highlighted by the correlation matrix, fruit firmness was negatively correlated with FWL (−0.744 ***), showing that progressive water loss was closely associated with tissue softening during storage. From a practical viewpoint, a higher dry matter content is linked to a lower proportion of free water, while increased firmness is generally associated with improved tolerance of vegetables to mechanical damage during postharvest handling and transport [35].

Fruit color, a key determinant of consumer perception, was mainly affected by ripening stage and storage time. These results are consistent with previous studies reporting limited or no effect of Se biofortification on tomato color parameters [23,25]. However, other authors have reported Se-dependent changes in color, often associated with delayed ripening due to partial inhibition of ethylene biosynthesis [20,34]. Such discrepancies may be explained by differences in genotype, ripening stage at harvest, biofortification protocols, and environmental conditions [26,36]. The observed changes during storage, including the decrease in lightness (L*) and variation in redness (a*), reflect the progression of pigment metabolism, with continued carotenoid synthesis in less mature fruits and degradation processes prevailing in fully ripe ones [37]. Indeed, L* values were highly negatively correlated with TCI (−0.934 *), confirming that increasing color intensity was accompanied by a marked reduction in lightness, while b* was positively correlated with both L* (0.671 ***) and a* (0.823 ***), but negatively correlated with TCI (−0.499 **). Concurrently, TCC was positively correlated with a* (0.339 *), confirming the well-known contribution of carotenoid accumulation to red color development.

Tomato sugars, mainly fructose and glucose, and organic acids content are the main compounds influencing the perceived tomato sweetness and sourness during mastication [38,39]. The increase in soluble sugars and the decrease in titratable acidity during ripening are tightly regulated at the transcriptional level, with key enzymes such as invertases and sucrose synthase involved in sugar metabolism, while organic acid degradation is promoted by specific enzymatic pathways [40,41,42]. Hormonal signals, particularly ethylene and abscisic acid, further regulate these processes [43,44]. In the present study, fruits harvested at the advanced ripening stage showed higher glucose, fructose, and total sugar contents, together with a lower titratable acidity; this resulted in a sweeter taste, as evidenced by the higher TSSs/TA ratio [42,45]. Se application influenced these traits in a ripening stage-dependent way. In particular, an increase in sugar contents was observed in fruits harvested at the deep red stage, whereas titratable acidity was higher in the Se-biofortified fruits harvested at earlier stages. These effects have been associated with the ability of Se to modulate ethylene production and respiration [20,21], potentially affecting the balance between sugar accumulation and acid degradation during ripening. Interestingly, the ”ripening stage × treatment” interaction revealed that although sugar levels declined over time, the Se-treated fruits harvested at stage G consistently maintained higher levels of glucose, fructose, and total sugars compared to the control. These results suggest that Se promoted a higher initial sugar loading at harvest and that this advantage was retained across the storage period. This finding is particularly relevant from a quality point of view, as a sweeter taste underpinned by higher sugar concentrations represents a pivotal quality attribute for cherry tomatoes, particularly when harvested at full ripeness [46]. Conversely, irrespective of storage duration, the Se-biofortified fruits harvested at stage E (i.e., those with lower initial carbohydrate levels) maintained a higher titratable acidity, indicating a delay in organic acid catabolism. Accordingly, in tomato, it has been reported that organic acids, particularly citrate, can act as alternative respiratory substrates under conditions of limited cytosolic carbohydrate availability [47]. Collectively, these findings support the hypothesis that Se modulates tomato postharvest behavior through the regulation of respiratory activity and associated hormonal metabolism [20].

Bioactive compounds, including phenolics, carotenoids, and ascorbic acid, are important contributors to tomato functional quality but are sensitive to postharvest variations [48]. In this study, total phenolic content was mainly influenced by ripening stage and storage time, with no significant effect of Se. Notably, TPC was negatively correlated with FWL (−0.572 **), TSC (−0.407 *), D-fructose (−0.358 *), and D-glucose (−0.442 **), and positively correlated with TA (0.502 **). These correlations suggest that phenolic retention could be favored in fruits with lower water loss and more limited metabolic advancement. Carotenoid content was affected by ripening stage and storage time, in agreement with previous studies describing a progressive increase during ripening [45,49]. In this case, the Se application reduced carotenoid content, particularly in fully ripe tomatoes. This was likely due to a Se-mediated delay in the chloroplast-to-chromoplast transition (typically hormone-driven) [50], likely determined by the partial suppression of ethylene biosynthesis, which would downregulate the transcriptional activation of key carotenoid biosynthesis genes. This finding represents an important trade-off which complicates the evaluation of Se biofortification outcomes in cherry tomatoes. Consequently, these results highlight the need for refined agronomic protocols (e.g., optimized doses, chemical forms, and application timing) to minimize potential adverse effects on the nutraceutical quality of the product. When considering carotenoid dynamics in stage E fruits during cold storage, these fruits showed a continuous increase in total carotenoid content (i.e., a condition of postharvest ripening), which, at S_20_, exceeded the levels observed in stage G fruits. Accordingly, carotenoid biosynthesis may continue during postharvest storage or transport, provided that cellular integrity is preserved, thereby maintaining the enzymatic activity involved in carotenogenesis [51,52]. The hypothesis of a protective role of Se on tomato cellular structures is further supported by the postharvest evolution of ascorbic acid content. Unlike control fruits, Se-biofortified tomatoes displayed a progressive increase in ascorbic acid levels throughout the storage period. This behavior is particularly relevant, as ascorbic acid, a six-carbon lactone, is highly susceptible to oxidative degradation after harvest and is widely regarded as a reliable indicator of overall quality deterioration during fruit and vegetable storage and handling [53]. Moreover, ascorbic acid represents a key nutraceutical compound in the human diet, owing to its essential role in antioxidant defense, immune function, and the prevention of chronic diseases [54,55]. Therefore, its accumulation in Se-treated fruits suggests not only an enhanced capacity to counteract oxidative stress, but also an improvement in the nutritional quality of the product. This response is consistent with previous findings [56] and supports the role of Se in strengthening antioxidant defense systems and preserving cellular integrity during postharvest storage. Interestingly, no significant correlations were observed between ascorbic acid and any of the other evaluated variables. Differently, the positive relationship between DPPH and TPC (0.667 ***) suggests that phenolic compounds represent a primary driver of radical scavenging capacity in these fruits, in line with the established role of polyphenols as major antioxidants in tomato [3].

## 4. Materials and Methods

### 4.1. Experimental Site, Plant Material, and Growth Conditions

The experiment was conducted on a greenhouse cherry tomato crop in Southern Italy (Sicily; 36°56′40″ N, 14°23′43″ E, 2 m a.s.l.). A multi-span, East–West oriented cold greenhouse was used, characterized by an overall area of 1000 m^2^ (50 × 20 m), lateral windows along the sides, and covered with an ethylene vinyl acetate film (200 μm thick, total visible transmission > 85%). The local climate is semi-arid Mediterranean, with mild winters and warm, rainless summers. The cropping period was selected to coincide with the typical autumn–winter production cycle of greenhouse cherry tomato in Mediterranean environments, thereby ensuring representative environmental conditions for commercial production. At the beginning of the experiment, the soil, classified as Entisol according to USDA Soil Taxonomy, had the following characteristics: 1.4 g kg^−1^ N, 6.3 g kg^−1^ organic matter, 12.1 mg kg^−1^ available P_2_O_5_, 74.6 mg kg^−1^ exchangeable K_2_O, and pH 7.6. The initial soil analysis indicated moderate fertility and slightly alkaline conditions, ensuring that plant growth and treatment responses were not limited by major soil nutritional constraints. The cherry tomato ‘Durillo’ (TSI Italia, Foggia, Italia) was chosen for the experiment, because of its spread over the reference area. On 7 September 2024, tomato seedlings at the four-leaf stage were transplanted in North–South oriented paired rows, with a center-to-center spacing of 2.00 m, and trained at single stem up to the 9th cluster. Within each pair, row spacing was 0.60 m, and the distance between plants within each row was 0.35 m (2.86 plants m^−2^). In the field, the experiment was arranged as a split-plot experimental design with three biological replicates, assigning the ripening stage at harvest to the main plots, and the foliar treatment to the subplots (see below). Each experimental unit included 24 plants (2 paired rows including 6 plants for each row, net of borders). Throughout the cropping cycle, plants were treated with two different foliar spray solutions: distilled water (control) vs. 0.5 mmol L^−1^ of Se as Na_2_SeO_4_ (hereafter Se). The selected concentration and Se form were based on previous studies demonstrating effective biofortification without phytotoxic effects [11]. All treatments were performed early in the morning (~8.00 a.m., local solar time) and started after the complete fruit set of the first cluster (~21 days after transplanting) and repeated after the fruit set of each cluster. In total, 9 treatments were applied (from 28 September 2024 to 20 January 2025), with each plant receiving ~7–25 mL solution per treatment (depending on growth stage). All solutions were supplied with 1 mL L^−1^ of a non-ionic surfactant (Vector^®^, Chimiberg, Caravaggio, BG, Italy) to enhance the adhesive properties. Drip irrigation (two drippers plant^−1^, 1.2 L h^−1^ each) was performed up to field capacity when the external accumulated evapotranspiration (calculated through the Penman–Monteith equation) reached 40 mm. Fertigation was performed by administering a complete nutrient solution [35], whereas pollination was promoted by using bumblebee hives. Further crop practices included the manual removal of lateral stems, and the application of *Beauveria bassiana* and Azadirachtin (when needed). All clusters were hand-harvested between 19th November and 28th February.

### 4.2. Fruit Sampling and Storage Conditions

Fruits from the 7th cluster (i.e., harvested between 7 and 12 February) were hand-harvested at two ripening stages, in accordance with commercial practice. According to Gautier et al. [45], these corresponded to stage E, defined as the stage at which the peduncular zone of at least eight proximal fruits per cluster exhibited a deep orange external coloration, and stage G, defined as the stage at which the peduncular zone of at least eight proximal fruits showed a deep red external coloration. Overall, 288 clusters (24 plants × 2 ripening stage × 2 Se treatments × 3 replicates) were divided into 3 main batches for the characterization of fruits after 0 (harvest date), 10, and 20 days of storage at 11.0 ± 0.5 °C and 80 ± 5% RH (vapor pressure deficit ~0.26 kPa, dew point 7.7 °C) (hereafter referred at S_0_, S_10_, and S_20_, respectively). To this end, fruits were detached from the rachis, selected for the absence of defects and uniformity, washed with deionized water and dried with paper towels. For characterization at S_10_ and S_20_, 20–25 fruits per replicate were placed in commercial PET trays (Mod. C500/41p; 190 × 115 × 41 mm) (Carton Pack s.p.a., Rutigliano, Italy) with a perforated lid for a final net weight of 250 ± 8 g, after which they were stored under the abovementioned conditions. Each experimental unit included 4 trays.

### 4.3. Determination of Main Carpometric Traits and Se Content

Soon after harvest, in order to detect any possible biofortification-induced alterations in visual appearance, fruit fresh weight (FW) was determined gravimetrically on a subset of 30 fruits per replicate using an electronic balance (accuracy ± 0.01 g). Additionally, the fruit shape index was calculated as the ratio of longitudinal to transverse diameter, measured with a digital caliper (CDJB15, Borletti, Novara, Italy) on 20 fruits per replicate. To determine the fruit Se content, 1 g of freeze-dried tomato sample was combined with 2 mL of ultrapure water and 2 mL of supra pure nitric acid. Digestion was aided by a microwave digestion system (ultraWAVE, Fkv, Bergamo, Italy). The samples were then transferred into polypropylene tubes and made up to a final volume of 50 mL with ultrapure water. The total Se content was quantified using an Agilent 7850 ICP-MS (Agilent Technologies, Santa Clara, CA, USA). Calibration was performed with a multi-element calibration standard solution (Agilent Technologies), and the limit of quantification was calculated based on the nine-fold standard deviation of a blank solution prepared and analyzed nine times.

At S_10_ and S_20_, fruit fresh weight loss (FWL) was assessed by reweighing the fruits within each tray. Fruit dry matter (DM) content was determined by reweighting a subsample of tomatoes (~150 g) after complete desiccation in a thermoventilated oven (Binder, Milan, Italy) at 105 °C. At each time point of storage, fruit firmness was measured through a digital texture analyzer (Stable Micro Systems, Godalming, UK) with a 490 N nominal force load cell and a stainless-steel plate as the probe (100 × 85 × 6 mm). All the tests were performed by applying a deformation force to the stylar end of each fruit (15 fruits per replicate) for up to 2 mm fruit deformation along the longitudinal axis, with a test speed of 10 mm s^−1^. The chromatic coordinates (CIE-L*a*b*) were measured along the fruit equatorial axis (2 measurements per fruit, 8 fruits per replicate), using a tristimulus Chroma Meter (CR-400, Konica Minolta, Tokyo, Japan) with illuminant D/65° and previously calibrated with a UE-certified standard white tile. Tomato color index (TCI) was calculated as reported by Distefano et al. [57].

### 4.4. Determination of Compositional Traits

For these determinations, at least twenty fruits per replicate were flash frozen using liquid nitrogen and freeze-dried until constant weight using a mod. Alpha 1–4 LD plus (Martin Christ, Osterode am Harz, Germany). Freeze-dried samples were then pulverized using a laboratory mill (A11 basic, IKA, Wilmington, NC, USA) and stored at −80 °C for further analyses. All further spectrophotometric analyses were performed using a UV-Vis spectrophotometer (mod. UV-1601, Shimadzu Corporation, Kyoto, Japan).

#### Determination of Sugars, Total Soluble Solids, and Titratable Acidity

Sucrose, D-glucose, and D-fructose contents were determined spectrophotometrically using the K-SUFRG Sucrose/D-Fructose/D-Glucose Enzymatic Assay Kit (Neogen, Lansing, MI, USA). Briefly, 500 mg of tomato powder was mixed with 25 mL of ultrapure water. The mixture was sonicated for 5 min in an ultrasonic bath, placed on an orbital shaker (IKA, Wilmington, NC, USA) at 180 rpm for 45 min, and then centrifugated (Neya 10R, Remi, Mumbai, India) for 5 min (4000× *g*). The resulting supernatant was collected and analyzed to determine the concentrations of the sugars following the manufacturer’s instructions. Since sucrose was detected only in trace amounts, total sugars content (TSC) was obtained by summing the contents of D-glucose and D-fructose. Results were expressed as mg g^−1^ dry weight (DW).

To determine the total soluble solids (TSSs) and titratable acidity (TA), at each sampling time, twenty representative fruits per replicate were blended using a home blender. The obtained puree was then centrifuged for 5 min (2376× *g*) to obtain a clear juice. Approximately 1–2 mL of clear juice was used to determine the TSSs via a digital refractometer (DBX-55, Atago, Tokyo, Japan) equipped with an automatic temperature compensation system. For the analysis of TA, 10 mL of tomato juice was titrated with 0.1 M NaOH to pH 8.2 by using phenolphthalein as an indicator until the pink end-point. Titratable acidity was calculated according to the equation reported by Sadler and Murphy [58], and results were expressed as % of citric acid equivalents (CAEs). The abovementioned determinations allowed for the calculation of the TSSs/TA ratio (adimensional).

### 4.5. Determination of Functional Traits

#### 4.5.1. Total Phenolic Content

Total phenolic content (TPC) of tomato was determined according to Cannata et al. [3] with minor adaptations. Briefly, 100 mg of freeze-dried sample were combined with 3 mL of methanol (80%), vortexed, and then sonicated for 10 min. Samples were then incubated for 15 min at 70 °C, after which the extracts were cooled on ice and centrifuged at 5345× *g* for 5 min (Neya 10R, Remi). An aliquot of 100 μL of supernatant was mixed with 700 μL of freshly prepared Folin–Ciocâlteu reagent (1:10 *v*/*v* in water); after 5 min, the solution was then mixed with 700 μL of Na_2_CO_3_ solution (6% *w*/*v*), gently agitated, and allowed to stand at room temperature for 1 h. The solution absorbance was read at 760 nm. Total phenolic content was calculated from a standard calibration curve (r = 0.999 ***) of gallic acid. The results were expressed as mg of gallic acid equivalents (GAE) g^−1^ DW.

#### 4.5.2. Total Carotenoids Content

Total carotenoid content (TCC, including xanthophylls) was determined according to Lichtenthaler and Buschmann [59], with adaptation. Briefly, 50 mg of lyophilized tomato powder was added to 6 mL acetone, briefly vortexed and followed by ultrasound assisted extraction (20 min at ~6 °C). The suspension was centrifuged (10 min, 5345× *g* at 6 °C; Neya 10R, Remi, Mumbai, India), and the supernatant transferred to glass vials. The solid phase was re-extracted using 6 mL acetone and the extracts were combined (total volume, 12 mL). The combined extracts were read spectrophotometrically at 470, 647, and 663 nm, and the obtained values were substituted in the standard equations reported by Lichtenthaler and Buschmann [59]. The results were expressed as µg g^−1^ DW.

#### 4.5.3. Ascorbic Acid Content

Total ascorbic acid (AsA) was determined as reported by Cannata et al. [58]. Briefly, 100 mg of freeze-dried sample was combined with 2 mL of H_3_PO_4_ (0.05 N) and then sonicated for 5 min and centrifugated (D3024R, Scilogex, Rocky Hill, CT, USA) for 15 min (20,000× *g*, at 4 °C). The supernatant was collected and filtered through a regenerated cellulose filter (0.45 μm, CHROMAFIL Xtra, Carlo Erba Reagents s.r.l., Milan, Italy) prior to analysis. The determination was performed using a Shimadzu High-Performance Liquid Chromatography (HPLC) system (Shimadzu Italia s.r.l., Milano, Italy) composed of two LC-20AD XR pumps, a SIL-40C XR auto sampler, and a SPD-M40 diode array detector. Separation at 30 °C was achieved using a SunShell C-18 analytic column (3.0 mm i.d. × 100 mm, 2.6 μm particle size, Lab Service analytica s.r.l., Bologna, Italy) protected by a guard column of the same material, and eluents consisted of 2% KH_2_PO_4_ buffer (pH 2.3) (B) and 100% acetonitrile (A). The applied elution gradient at a constant flow rate of 0.5 mL min^−1^ was isocratic at 98% B (1 min), from 98% to 88% B (7 min), 0 to 98% B (2 min), and isocratic hold at 98% B (1 min). Total run time was 11 min, and the injection volume was 10 µL. Spectra were recorded between 190 and 400 nm, and AsA was detected at 245 nm. A liner calibration curve of AsA standard (Fisher Scientific Italia, Milan, Italy) was established before the sample analysis. Results were reported as mg g^−1^ DW.

#### 4.5.4. 2,2-Diphenyl-1-picrylhydrazyl Assay

The 2,2-diphenyl-1-picrylhydrazyl (DPPH) radical scavenging activity of tomatoes was determined according to Brand–Williams et al. [60] with modifications. Specifically, 100 mg of lyophilized tomato powder was combined with 3 mL methanol (80%), placed into an ultrasonic bath (10 min at ~6 °C), and centrifuged for 15 min (2236× *g* at 6 °C). Subsequently, the supernatant was collected and the decrease in absorbance of the methanolic solution was read spectrophotometrically at 515 nm. The values were then calculated from a standard calibration curve (r = 0.998 ***) obtained by plotting the change in absorbance against different Trolox concentrations. Results were expressed as mg Trolox equivalents (TEs) g^−1^ DW.

### 4.6. Statistical Procedures

Fruit FW, L, D, and Se content were recorded only at harvest, and hence were analyzed using a two-way analysis of variance (ANOVA, ripening stage × treatment) according to the design adopted in the field. The remaining traits were subjected to a three-way ANOVA (ripening stage × treatment × storage time). Prior analysis, the obtained data were subjected to Shapiro–Wilk’s and Levene’s tests, to check for normal distribution and homoscedasticity, respectively. Percentage values were subjected to Bliss transformation before the ANOVA (untransformed data are reported). Post hoc comparisons among means were performed using Tukey’s Honestly Significant Difference (HSD) test (*p* ≤ 0.05). Pearson’s correlation analysis was performed on the pooled dataset including all experimental observations across ripening stages, treatments, and storage times, in order to explore overall associations among the measured variables across the entire experimental dataset. All calculations were conducted using Excel version 2016 (Microsoft Corporation, Redmond, WA, USA) and Jamovi software (version 2.3.5), and Excel VBA add-in DSAASTAT [61].

## 5. Conclusions

Overall, the results of the present experiment highlight the complex effects of preharvest Se application on cherry tomatoes, from the dual perspective of (i) enhancing their nutrient density and (ii) improving their postharvest quality evolution during refrigerated storage. From the first perspective, the most relevant finding is the marked increase in Se content of the product (~30-fold), which was not influenced by the ripening stage. This suggests sustained Se uptake and translocation kinetics to the fruits, making the outcome of potential biofortification programs reliable and unaffected by the harvest timing. Moreover, our results demonstrate that this strong mineral enrichment occurred without any alteration of the main morphometric traits of the product, which could otherwise affect consumer perception of biofortified tomatoes. From the second perspective, the increased dry matter content of the fruits (and the greater stability of this parameter during storage), together with the higher firmness observed in biofortified tomatoes, suggest tangible benefits in terms of improved postharvest performance and extended marketable shelf life. From a compositional standpoint, several key quality parameters were improved during storage in response to Se biofortification, although these responses were dependent on the ripening stage at harvest. Specifically, in fruits harvested at the deep red stage, Se enhanced the preservation of soluble sugars during storage, indicating improved maintenance of carbohydrate status. However, Se application reduced carotenoid content in fully ripe tomatoes. In contrast, Se promoted the accumulation of ascorbic acid and enhanced its retention during storage, regardless of ripening stage.

Overall, these results highlight the contrasting, stage-dependent role of Se biofortification in strengthening the functional profile and preserving the nutritional quality of cherry tomatoes under refrigerated conditions. Taken together, these findings emphasize the need to refine the biofortification strategy in order to achieve a more consistent and comprehensive improvement in fruit quality, with the ripening stage at harvest emerging as a key determinant in optimizing the overall effectiveness of Se biofortification.

## Data Availability

The data presented in this study are available on request from the corresponding author.

## Abbreviations

The following abbreviations are used in this manuscript:

ANOVA	Analysis of variance
AsA	Ascorbic acid
DM	Dry matter
FW	Fresh weight
FWL	Fresh weight loss
HSD	Honestly significant difference
ROS	Reactive oxygen species
TA	Titratable acidity
TCI	Tomato color index
TCC	Total carotenoid content
TPC	Total phenolic content
TSS	Total soluble solids
TSC	Total sugars content

## References

[1] FAOSTAT. https://www.fao.org/faostat/en/#data/QCL.

[2] ISTAT Superfici e Produzione—Dati in Complesso. https://esploradati.istat.it/databrowser/#/it.

[3] Cannata C., Mauro R.P., Rutigliano C.A.C., Basile F., Muratore G., Restuccia C., Sabatino L., Leonardi C. (2024). Exploring the Evolution of Postharvest Quality and Composition in Novel Mini Plum Tomatoes with Different Fruit Pigmentations. Agronomy.

[4] Yadav A., Kumar N., Upadhyay A., Sethi S., Singh A. (2022). Edible Coating as Postharvest Management Strategy for Shelf-life Extension of Fresh Tomato (*Solanum lycopersicum* L.): An Overview. J. Food Sci..

[5] Rutigliano C.A.C., Cannata C., Restuccia C., Mauro R.P., Muratore G., Leonardi C. (2025). Eco-Friendly Packaging System for Ready-to-Eat Tomatoes. Acta Hortic..

[6] Karapanos I.C., Chandra M., Akoumianakis K.A., Passam H.C., Alexopoulos A.A. (2015). The Ripening and Quality Characteristics of Cherry Tomato Fruit in Relation to the Time of Harvest. Acta Hortic..

[7] Duret S., Aubert C., Annibal S., Derens-Bertheau E., Cottet V., Jost M., Chalot G., Flick D., Moureh J., Laguerre O. (2025). Impact of Harvest Maturity and Storage Conditions on Tomato Quality: A Comprehensive Experimental and Modeling Study. Postharvest Biol. Technol..

[8] Khalid S., Hassan S.A., Javaid H., Zahid M., Naeem M., Bhat Z.F., Abdi G., Aadil R.M. (2024). Factors Responsible for Spoilage, Drawbacks of Conventional Packaging, and Advanced Packaging Systems for Tomatoes. J. Agric. Food Res..

[9] Di Giacomo M., Luciani M.D., Cambiaso V., Zorzoli R., Rodríguez G.R., Pereira da Costa J.H. (2020). Tomato near Isogenic Lines to Unravel the Genetic Diversity of S. Pimpinellifolium LA0722 for Fruit Quality and Shelf Life Breeding. Euphytica.

[10] Bjørklund G., Shanaida M., Lysiuk R., Antonyak H., Klishch I., Shanaida V., Peana M. (2022). Selenium: An Antioxidant with a Critical Role in Anti-Aging. Molecules.

[11] Cannata C., Giuffrida F., Sabatino L., Basile F., Vultaggio L., Leonardi C., Mauro R.P. (2025). Selenium Biofortification of Greenhouse Cherry Tomato: Impact on Fruit Quality Traits and Selenium Content. Acta Hortic..

[12] Zhu Z., Zhang Y., Liu J., Chen Y., Zhang X. (2018). Exploring the Effects of Selenium Treatment on the Nutritional Quality of Tomato Fruit. Food Chem..

[13] Consentino B.B., Mancuso F., Vultaggio L., Bellitto P., Ntatsi G., Cannata C., La Placa G.G., Mauro R.P., La Bella S., Sabatino L. (2026). Selenium Biofortification and an Ecklonia Maxima-Based Seaweed Extract Jointly Compose Curly Endive Drought Stress Tolerance in a Soilless System. Plants.

[14] Agostoni C., Berni Canani R., Fairweather-Tait S., Heinonen M., Korhonen H., La Vieille S., Marchelli R., Martin A., Naska A., Neuhäuser-Berthold M. (2014). Scientific Opinion on Dietary Reference Values for Selenium. EFSA J..

[15] Leonardi C., Cannata C., Giuffrida F., Basile F., Fichera G., Mauro R.P. (2025). Agronomic Mineral Biofortification to Enhance the Nutritional Value of Vegetables: A Review. Acta Hortic..

[16] Liu L., Luo P., Wen P., Xu P. (2024). Effects of Selenium and Iodine on Kashin-Beck Disease: An Updated Review. Front. Nutr..

[17] Jones G.D., Droz B., Greve P., Gottschalk P., Poffet D., McGrath S.P., Seneviratne S.I., Smith P., Winkel L.H.E. (2017). Selenium Deficiency Risk Predicted to Increase under Future Climate Change. Proc. Natl. Acad. Sci. USA.

[18] Schiavon M., Dall’Acqua S., Mietto A., Pilon-Smits E.A.H., Sambo P., Masi A., Malagoli M. (2013). Selenium Fertilization Alters the Chemical Composition and Antioxidant Constituents of Tomato (*Solanum lycopersicon* L.). J. Agric. Food Chem..

[19] Zhu Z., Chen Y., Zhang X., Li M. (2016). Effect of Foliar Treatment of Sodium Selenate on Postharvest Decay and Quality of Tomato Fruits. Sci. Hortic..

[20] Puccinelli M., Malorgio F., Terry L.A., Tosetti R., Rosellini I., Pezzarossa B. (2018). Effect of Selenium Enrichment on Metabolism of Tomato (*Solanum lycopersicum*) Fruit during Postharvest Ripening. J. Sci. Food Agric..

[21] Zhu Z., Chen Y., Shi G., Zhang X. (2017). Selenium Delays Tomato Fruit Ripening by Inhibiting Ethylene Biosynthesis and Enhancing the Antioxidant Defense System. Food Chem..

[22] Domínguez E., Fernández M.D., Hernández J.C.L., Parra J.P., España L., Heredia A., Cuartero J. (2012). Tomato Fruit Continues Growing While Ripening, Affecting Cuticle Properties and Cracking. Physiol. Plant..

[23] de Morais E.G., Silva M.A., Quispe A.P.V., Machado G.G.L., Prado D.T., Benevenute P.A.N., Lima J.d.S., de Sousa G.F., de Barros Vilas Boas E.V., Guilherme L.R.G. (2024). Foliar Sprays of Multi-Nutrient Fertilizer Containing Selenium Produce Functional Tomato Fruits with Higher Shelf Life. Plants.

[24] Neysanian M., Iranbakhsh A., Ahmadvand R., Oraghi Ardebili Z., Ebadi M. (2020). Comparative Efficacy of Selenate and Selenium Nanoparticles for Improving Growth, Productivity, Fruit Quality, and Postharvest Longevity through Modifying Nutrition, Metabolism, and Gene Expression in Tomato; Potential Benefits and Risk Assessment. PLoS ONE.

[25] Shiriaev A., Pezzarossa B., Rosellini I., Malorgio F., Lampis S., Ippolito A., Tonutti P. (2022). Efficacy and Comparison of Different Strategies for Selenium Biofortification of Tomatoes. Horticulturae.

[26] Izydorczyk G., Ligas B., Mikula K., Witek-Krowiak A., Moustakas K., Chojnacka K. (2021). Biofortification of Edible Plants with Selenium and Iodine—A Systematic Literature Review. Sci. Total Environ..

[27] Xu X., Wang J., Wu H., Yuan Q., Wang J., Cui J., Lin A. (2022). Effects of Selenium Fertilizer Application and Tomato Varieties on Tomato Fruit Quality: A Meta-Analysis. Sci. Hortic..

[28] Turck D., Bohn T., Castenmiller J., de Henauw S., Hirsch-Ernst K., Knutsen H.K., Maciuk A., Mangelsdorf I., McArdle H.J., Peláez C. (2023). Scientific Opinion on the Tolerable Upper Intake Level for Selenium. EFSA J..

[29] Brummell D.A., Harpster M.H. (2001). Cell Wall Metabolism in Fruit Softening and Quality and Its Manipulation in Transgenic Plants. Plant Mol. Biol..

[30] Dorairaj D., Sharma S., Mawale K.S., Puthusseri B., Parvatam G., Shetty N.P. (2025). Determining the Function of Ripening Associated Genes and Biochemical Changes during Tomato (*Solanum lycopersicum* L.) Fruit Maturation. Biotechnol. Lett..

[31] Díaz-Pérez J.C. (2019). Transpiration. Postharvest Physiology and Biochemistry of Fruits and Vegetables.

[32] Guillén F., Castillo S., Zapata P.J., Martínez-Romero D., Valero D., Serrano M. (2006). Efficacy of 1-MCP Treatment in Tomato Fruit. Postharvest Biol. Technol..

[33] Jalali P., Roosta H.R., Khodadadi M., Torkashvand A.M., Jahromi M.G. (2022). Effects of Brown Seaweed Extract, Silicon, and Selenium on Fruit Quality and Yield of Tomato under Different Substrates. PLoS ONE.

[34] Pezzarossa B., Rosellini I., Borghesi E., Tonutti P., Malorgio F. (2014). Effects of Se-Enrichment on Yield, Fruit Composition and Ripening of Tomato (*Solanum lycopersicum*) Plants Grown in Hydroponics. Sci. Hortic..

[35] Cannata C., Basile F., Mauro R.P., Giordano M., Susino M.C., Leonardi C. (2023). Variegated Bioactive Potential and Different Productive Responses Displayed by a Set of Polychromatic Mini Plum Tomato Cultivars. Italus Hortus.

[36] Xu X., Ye S., Zuo X., Fang S. (2022). Impact of Guar Gum and Locust Bean Gum Addition on the Pasting, Rheological Properties, and Freeze–Thaw Stability of Rice Starch Gel. Foods.

[37] López Camelo A.F., Gómez P.A. (2004). Comparison of Color Indexes for Tomato Ripening. Hortic. Bras..

[38] Bertin N., Génard M. (2018). Tomato Quality as Influenced by Preharvest Factors. Sci. Hortic..

[39] Oltman A.E., Jervis S.M., Drake M.A. (2014). Consumer Attitudes and Preferences for Fresh Market Tomatoes. J. Food Sci..

[40] Pesaresi P., Mizzotti C., Colombo M., Masiero S. (2014). Genetic Regulation and Structural Changes during Tomato Fruit Development and Ripening. Front. Plant Sci..

[41] Quinet M., Angosto T., Yuste-Lisbona F.J., Blanchard-Gros R., Bigot S., Martinez J.-P., Lutts S. (2019). Tomato Fruit Development and Metabolism. Front. Plant Sci..

[42] Beckles D.M. (2012). Factors Affecting the Postharvest Soluble Solids and Sugar Content of Tomato (*Solanum lycopersicum* L.) Fruit. Postharvest Biol. Technol..

[43] Liu M., Pirrello J., Chervin C., Roustan J.P., Bouzayen M. (2015). Ethylene Control of Fruit Ripening: Revisiting the Complex Network of Transcriptional Regulation. Plant Physiol..

[44] Zhang M., Yuan B., Leng P. (2009). The Role of ABA in Triggering Ethylene Biosynthesis and Ripening of Tomato Fruit. J. Exp. Bot..

[45] Gautier H., Diakou-Verdin V., Bénard C., Reich M., Buret M., Bourgaud F., Poëssel J.L., Caris-Veyrat C., Génard M. (2008). How Does Tomato Quality (Sugar, Acid, and Nutritional Quality) Vary with Ripening Stage, Temperature, and Irradiance?. J. Agric. Food Chem..

[46] Cannata C., Steingass C.B., May B., Schweiggert R., Rouphael Y., Leonardi C., Giuffrida F., Mauro R.P. (2026). Genotype and Cluster Position Influence Carotenoid and Mineral Profiles of Mini Plum Tomatoes in Response to Zinc Biofortification and Triacontanol Application. J. Agric. Food Res..

[47] Etienne A., Génard M., Lobit P., Mbeguié-A-Mbéguié D., Bugaud C. (2013). What Controls Fleshy Fruit Acidity? A Review of Malate and Citrate Accumulation in Fruit Cells. J. Exp. Bot..

[48] Pott D.M., Vallarino J.G., Osorio S. (2020). Metabolite Changes during Postharvest Storage: Effects on Fruit Quality Traits. Metabolites.

[49] Raffo A., Leonardi C., Fogliano V., Ambrosino P., Salucci M., Gennaro L., Bugianesi R., Giuffrida F., Quaglia G. (2002). Nutritional Value of Cherry Tomatoes (*Lycopersicon esculentum* Cv. Naomi F1) Harvested at Different Ripening Stages. J. Agric. Food Chem..

[50] Cruz A.B., Bianchetti R.E., Alves F.R.R., Purgatto E., Peres L.E.P., Rossi M., Freschi L. (2018). Light, Ethylene and Auxin Signaling Interaction Regulates Carotenoid Biosynthesis during Tomato Fruit Ripening. Front. Plant Sci..

[51] Ngamwonglumlert L., Devahastin S., Chiewchan N., Raghavan V. (2020). Plant Carotenoids Evolution during Cultivation, Postharvest Storage, and Food Processing: A Review. Compr. Rev. Food Sci. Food Saf..

[52] Orsi B., Sestari I., Preczenhak A.P., Tessmer M.A., da Silva Souza M.A., Hassimotto N.M.A., Kluge R.A. (2021). Allelic Variations in the Tomato Carotenoid Pathway Lead to Pleiotropic Effects on Fruit Ripening and Nutritional Quality. Postharvest Biol. Technol..

[53] Giannakourou M.C., Taoukis P.S. (2021). Effect of Alternative Preservation Steps and Storage on Vitamin C Stability in Fruit and Vegetable Products: Critical Review and Kinetic Modelling Approaches. Foods.

[54] Alberts A., Moldoveanu E.T., Niculescu A.G., Grumezescu A.M. (2025). Vitamin C: A Comprehensive Review of Its Role in Health, Disease Prevention, and Therapeutic Potential. Molecules.

[55] Ali A., Riaz S., Khalid W., Fatima M., Mubeen U., Babar Q., Manzoor M.F., Zubair Khalid M., Madilo F.K. (2024). Potential of Ascorbic Acid in Human Health against Different Diseases: An Updated Narrative Review. Int. J. Food Prop..

[56] Islam S., Mohammad F. (2020). Triacontanol as a Dynamic Growth Regulator for Plants under Diverse Environmental Conditions. Physiol. Mol. Biol. Plants.

[57] Distefano M., Cincotta F., Giuffrida F., Condurso C., Sabatino L., Verzera A., Leonardi C., Cannata C., Consentino B.B., Mauro R.P. (2025). Effects of Preharvest Monopotassium Phosphate Applications on Tomato Fruit Quality during Cold Storage. Acta Hortic..

[58] Cannata C., Rutigliano C.A.C., Restuccia C., Muratore G., Sabatino L., Geoffriau E., Leonardi C., Mauro R.P. (2025). Effects of Polysaccharide-Based Edible Coatings on the Shelf Life of Fresh-Cut Carrots with Different Pigmentations. J. Agric. Food Res..

[59] Lichtenthaler H.K., Buschmann C. (2001). Chlorophylls and Carotenoids: Measurement and Characterization by UV-VIS Spectroscopy. Curr. Protoc. Food Anal. Chem..

[60] Brand-Williams W., Cuvelier M.E., Berset C. (1995). Use of a Free Radical Method to Evaluate Antioxidant Activity. LWT—Food Sci. Technol..

[61] Onofri A. Routine Statistical Analyses of Field Experiments by Using an Excel^®^ Extension. Proceedings of the 6th National Conference Italian Biometric Society: “La Statistica nelle Scienze della vita e Dell’ambiente”.

